# A comparison of statistical methods for the detection of hepatocellular carcinoma based on serum biomarkers and clinical variables

**DOI:** 10.1186/1755-8794-6-S3-S9

**Published:** 2013-11-11

**Authors:** Mengjun Wang, Anand Mehta, Timothy M Block, Jorge Marrero, Adrian M Di Bisceglie, Karthik Devarajan

**Affiliations:** 1 3508 Old Easton Rd, Doylestown, PA, 18902, USA; 2 Division of Gastroenterology, University of Michigan, 3912 Taubman Center, Ann Arbor, MI 48109, USA; 3 Saint Louis University School of Medicine, 1402 S. Grand FDT 12th Floor, St. Louis, MO 63104, USA; 4 Department of Biostatistics and Bioinformatics, Fox Chase Cancer Center, 333 Cottman Avenue, Philadelphia, PA 18901, USA

## Abstract

**Background:**

Currently, a surgical approach is the best curative treatment for those with hepatocellular carcinoma (HCC). However, this requires HCC detection and removal of the lesion at an early stage. Unfortunately, most cases of HCC are detected at an advanced stage because of the lack of accurate biomarkers that can be used in the surveillance of those at risk. It is believed that biomarkers that could detect HCC early will play an important role in the successful treatment of HCC.

**Methods:**

In this study, we analyzed serum levels of alpha fetoprotein, Golgi protein, fucosylated alpha-1-anti-trypsin, and fucosylated kininogen from 113 patients with cirrhosis and 164 serum samples from patients with cirrhosis plus HCC. We utilized two different methods, namely, stepwise penalized logistic regression (*stepPLR*) and model-based classification and regression trees (*mob*), along with the inclusion of clinical and demographic factors such as age and gender, to determine if these improved algorithms could be used to increase the detection of cancer.

**Results and discussion:**

The performance of multiple biomarkers was found to be better than that of individual biomarkers. Using several statistical methods, we were able to detect HCC in the background of cirrhosis with an area under the receiver operating characteristic curve of at least 0.95. *stepPLR *and *mob *demonstrated better predictive performance relative to logistic regression (LR), penalized LR and classification and regression trees (CART) used in our prior study based on three-fold cross-validation and leave one out cross-validation. In addition, *mob *provided unparalleled intuitive interpretation of results and potential cut-points for biomarker levels. The inclusion of age and gender improved the overall performance of both methods among all models considered, while the stratified male-only subset provided the best overall performance among all methods and models considered.

**Conclusions:**

In addition to multiple biomarkers, the incorporation of age and gender into statistical models significantly improved their predictive performance in the detection of HCC.

## Background

The major etiology of hepatocellular carcinoma is infection with hepatitis B virus (HBV) and/or hepatitis C virus (HCV) [1-5], which can lead to liver cirrhosis, the main risk factor for HCC. Worldwide, it is estimated that between 500,000-700,000 people die as a result of HCC every year [2,5,7].

Surgical treatments, such as tumor ablation, resection and transplantation still offer the best hope for long term survival but work best when tumors are caught at an early stage. Thus, the screening of the cirrhotic patient population for early detection is thought to be an important step to increase survival.

Currently, patients at risk for HCC are monitored either by imaging and/or through the use by serum levels of the glycoprotein, alpha-fetoprotein (AFP) or the core fucosylated glycoform of AFP (AFP-L3). However, AFP can have poor sensitivity and specificity [8-10], and is not present in many patients with HCC. Therefore the use of AFP as the primary screen for HCC is questioned [11] and more specific and sensitive, serum biomarkers for HCC are urgently needed [12-16].

We have previously observed increased levels of fucosylated proteins in the serum of those with HCC and through the use of fucose specific lectins we identified many of the proteins that become fucosylated with liver disease [17-19]. In the current study we have analyzed the performance of several of these potential biomarkers in the serum from 113 patients with cirrhosis and 164 serum samples from patients with cirrhosis plus HCC. In an effort to maximize the detection of patients with cancer, we applied several novel bio-statistical tools to determine if improved algorithms would aid in the detection of cancer. This included combining biomarker values with clinical and demographic factors such as age and gender to improve diagnosis. Using several of these methods, we are able to detect HCC in the background of cirrhosis with a predictive probability of at least 0.95, a significant improvement relative to that of any marker when used alone. The potential benefit of using this combination of markers and clinical variables is discussed in this paper.

## Methods

### Patients

Serum samples were obtained from Saint Louis University School of Medicine or the University of Michigan. For samples obtained from the University of Michigan, the study protocol was approved by the University of Michigan's Institutional Review Board and written informed consent was obtained from each subject. Demographic and clinical information was obtained, and a blood sample was collected from each subject. Patients with HCC, and patients with cirrhosis that were age, gender, and race/ethnicity matched to the HCC patients were enrolled from the Liver Clinic during this period. The diagnosis of HCC was made by histopathology, including all T1 lesions, and if histopathology was NA by two imaging modalities (ultrasound [US], magnetic resonance imaging [MRI], or computed tomography) showing a vascular enhancing mass > 2 cm) [5]. Diagnosis of cirrhosis was based on liver histology or clinical, laboratory and imaging evidence of hepatic de-compensation or portal hypertension [15]. Each of the patients with cirrhosis had a normal US and, if serum AFP was elevated, a MRI of the liver within 3 months prior to enrollment and another one 6 months after enrollment that showed no liver mass. The cirrhotic controls have been followed for a median of 12 months (range 7-18 months) after enrollment, and no one has developed HCC. A 20-ml blood sample was drawn from each subject, spun, aliquoted, and serum stored at -80°C until testing. Blood samples were drawn prior to initiation of HCC treatment. AFP was tested using commercially available immunoassays utilizing enhanced chemiluminescence at the University of Michigan Hospital Clinical Diagnostic Laboratory. The upper limit of normal was 8 ng/ml.

For samples obtained from Saint Louis University School of Medicine, the study protocol was approved by the Saint Louis University Institutional Review Board and written informed consent was obtained from each subject. Demographic and clinical information was obtained, and a blood sample was collected from each subject in a serum separator tube, spun within 2 hours and serum stored at -80°C until testing. Subjects either had HCC on biopsy, a new hepatic defect showing vascular enhancement on one imaging modality (ultrasound [US], magnetic resonance imaging [MRI], or computed tomography [CT]) with AFP > 1000 ng/ml or presumed HCC. Subjects were presumed to have HCC if they had a discrete hepatic defect on US with AFP < 1000 ng/ml and either 2 other scans (MRI, CT, angiography) indicating malignancy with at least 1 of the following characteristics: Hypervascularity; arterial to portal vein shunts, portal vein thrombosis near the defect, tumor in the portal vein or 1 other scan (MRI or CT) showing features characteristic of HCC and either an increase in size over time after initial discovery (at least doubling if less than 1 cm) or an increase in AFP to > 200 ng/ml. For the cirrhosis group, patients with Hepatitis C and biopsy proven cirrhosis were enrolled. All cirrhotic controls were screened for HCC using US, CT or MRI prior to enrollment.

### Lectin FLISA

Monoclonal antibodies are fucosylated and are reactive with fucose binding lectins. Hence they must be modified prior to analysis via the Lectin-FLISA. Briefly, to remove the fucosylation of the capture antibody (Mouse anti-human A1AT or rabbit anti-human LMW kininogen, Bethyl Laboratories, Montgomery, TX), antibody was incubated with 10mM sodium periodate for 1 hour at 37°C. An equal volume of ethylene glycol was added and the oxidized antibody brought to a concentration of 10 μg/mL with sodium carbonate buffer, pH 9.5. Antibody (1 μg/well) was added to the plate and following incubation washed with 0.1% Tween 20/PBS 7.4 and blocked overnight with 3% BSA/PBS. For analysis, 5 μl of serum was diluted in 95 μL of Heterophilic Blocking tubes (Scantibodies Laboratory, Inc. Santee, CA 92071 USA) and incubated at room temperature for 1 hour. Subsequently, samples were added to the plates for 2 hours and washed 5 times in lectin incubation buffer (10mM Tris pH 8.0, 0.15M NaCl, 0.1%Tween 20) before fucosylated protein was detected with a biotin conjugated *Aleuria aurantia *(*AAL*) lectin (Vector Laboratories, Burlingame, CA). Bound lectin was detected using IRDye™ 800 Conjugated streptavidin and signal intensity measured using the Odyssey^® ^Infrared Imaging System (LI-COR Biotechnology, Lincoln, Nebraska) as described in [20,21]. In all cases sample intensity was compared to commercially purchased human serum (Sigma Inc., St Louis, MO.).

### Immunoblotting for GP73

Equal volumes of patient sera were resolved by SDS-PAGE on 10% polyacrylamide gels and the proteins transferred to a PVDF membrane by immunoblotting. The membranes were blocked by incubating with a blocking buffer of 1x TBS (50 mM Tris-HCl, pH 7.6, 150 mM sodium chloride), 5% non-fat dried milk, and 0.1% Tween 20 for 1 hour at room temperature. The blots were incubated overnight with polyclonal anti-GP73 antibody (1:2000) and incubated with rocking at room temperature for 2 hours. Blots were subsequently washed 3x10mins 0.1% Tween-PBS and GP73 visualized using an IRDye™ 700 Conjugated mouse anti-rabbit secondary antibody (1:10,000). Signal intensity measured using the Odyssey^® ^Infrared Imaging System (LI-COR Biotechnology, Lincoln, Nebraska). In all cases sample intensity was compared to commercially purchased human serum (Sigma Inc., St Louis, MO).

### Statistical methods

Univariate statistical analyses were performed using Fisher's exact test for categorical variables and the Mann-Whitney test for continuous variables. Univariate logistic regression analyses were also performed for each individual biomarker separately. Details of univariate analysis results are presented in [26]. A variety of methods and models were used in multivariable analyses for associating the incidence of HCC with biomarker levels and clinical/demographic variables such as age and gender. Specifically, two different but related methods were investigated in this approach - stepwise PLR (*stepPLR*) and model-based CART (*mob*). These two methods are improvisations of PLR and CART described in our previous work [26]. A variety of models were considered for each method. Details of these methods are provided in the ensuing paragraphs. All tests were two-sided and used a Type I Error of 0.05 to determine statistical significance.

PLR is a variant of logistic regression based on a quadratic penalty that is ideal for associating discrete factors and continuous variables such as gender, age and biomarker levels with a binary response such as HCC incidence. In PLR, we maximize the log-likelihood subject to a size constraint on the *L*_2_-norm of the coefficients (excluding the intercept) [22]. This penalized likelihood can be written as

$$L\left(\beta_{0},\beta ,\lambda \right)=l\left(\beta_{0},\beta \right)-\frac{\lambda }{2}||\beta |{|}_{2}^{2}$$

where *l *indicates the binomial log-likelihood and λ is a positive constant. The use of quadratic penalization provides stability to the model fit by overcoming collinearity among variables. Even though the number of variables in our application is limited, PLR is well suited for modeling a large number of variables. The sample size does not limit the number of such variables, and variable selection can be done using a forward stepwise approach. PLR is implemented in the open-source R package stepPLR (http://www.r-project.org) [23]. The standard PLR approach is applied to a fixed set of biomarkers, clinical and/or demographic variables and was used in our previous work [26]. We extended this method to incorporate stepwise model selection in this paper. *stepPLR *provides the functionality for stepwise model selection based on PLR for a fixed value of λ. It tests for interactions between biomarkers, demographic and clinical variables and removes all non-significant terms. Stepwise regression is then performed for the pre-specified λ, and the remaining significant terms are included in the final model. stepPLR is typically repeated for various pre-specified values of λ and the best performing model is chosen. Three different values of the penalty parameter λ (0.1,1,10) were considered in our approach.

CART is based on decision trees and is a non-parametric approach. A decision tree is a logical model represented as a binary tree that shows how the value of a response variable can be predicted by using the values of a set of clinical variables. If the response variable is binary such as whether a patient developed HCC or not, then a classification tree is generated that predicts the probability of developing HCC. The unified CART framework based on conditional inference trees embeds recursive binary partitioning into the theory of permutation tests [24]. This methodology is applicable to all types of regression settings and overcomes the problem of over-fitting and selection bias towards variables with many possible splits or missing values. The conditional distribution of statistics used in this approach results in unbiased selection among covariates measured at different scales. Significance testing procedures are applied to determine whether no significant association between any of the covariates and the response can be stated and the recursion needs to stop. The function ctree() in the open-source R package PARTY (http://www.r-project.org) [25] implements this non-parametric approach and was used in our previous work [26]. In this paper, we extended this approach to incorporate parametric modeling. Specifically, it borrows strength from binary recursive partitioning in CART and the parametric approach in LR. This model-based approach to CART is based on generalized linear models [24] and is implemented in the function mob() of the package PARTY. It was used to model the effects of biomarker levels, gender and age associated with the development of HCC.

In order to evaluate the performance of statistical models combining multiple biomarkers and/or clinical variables, values of multiple biomarkers were inputted into the model from the appropriate method, and in each case the output (predicted value) was between 0 and 1, with 0 being cirrhosis and 1 being cancer. A cut-off of 0.5 was used for the predicted probability *p *and patients were classified as being HCC positive when *p *>= 0.5, otherwise they were classified as cirrhotic (*p*<0.5). To determine the optimal cutoff value for each biomarker or a combination of biomarkers and/or clinical variables, Receiver Operating Characteristic (ROC) curves were constructed using all possible cutoffs for each method. Sensitivity and specificity (along with 95% confidence interval (CI)) were used to characterize the precision of binary predictions from *stepPLR *and *mob*. Area under the ROC curves (AUC) (along with 95% CI), prediction accuracy (ACC) positive predictive value (PPV) and negative predictive value (NPV) were used to characterize the predictive value of models from these methods. For each model considered, the Akaike Information Criterion (AIC) was calculated.

In addition, the performance of each model was evaluated using leave-one-out cross validation (LOOCV) and three-fold cross validation (3CV). For details on LOOCV and 3CV, the interested reader is referred to our previous work [26]. Using results from LOOCV, an ROC curve and its AUC (with 95% CI) was computed based on the predicted probabilities. This is the cross-validated AUC. Likewise, sensitivities at set specificities from this ROC curve can be estimated. In order to evaluate the performance of each model on independent data in the absence of a validation set, 3CV was used. Using 200 random partitions of the dataset based on 3CV, the mean AUC, its standard deviation and 95% CI were computed.

## Results and discussion

### Univariate analysis

A significant association between gender and the incidence of HCC was found, with a significantly increased odds of HCC in males (odds ratio = 1.75) compared to females. A statistically significant association between age and incidence of HCC was also observed. Results of univariate analyses are reported in detail in our previous study [26].

### Multivariable analysis

**Figure 1:**
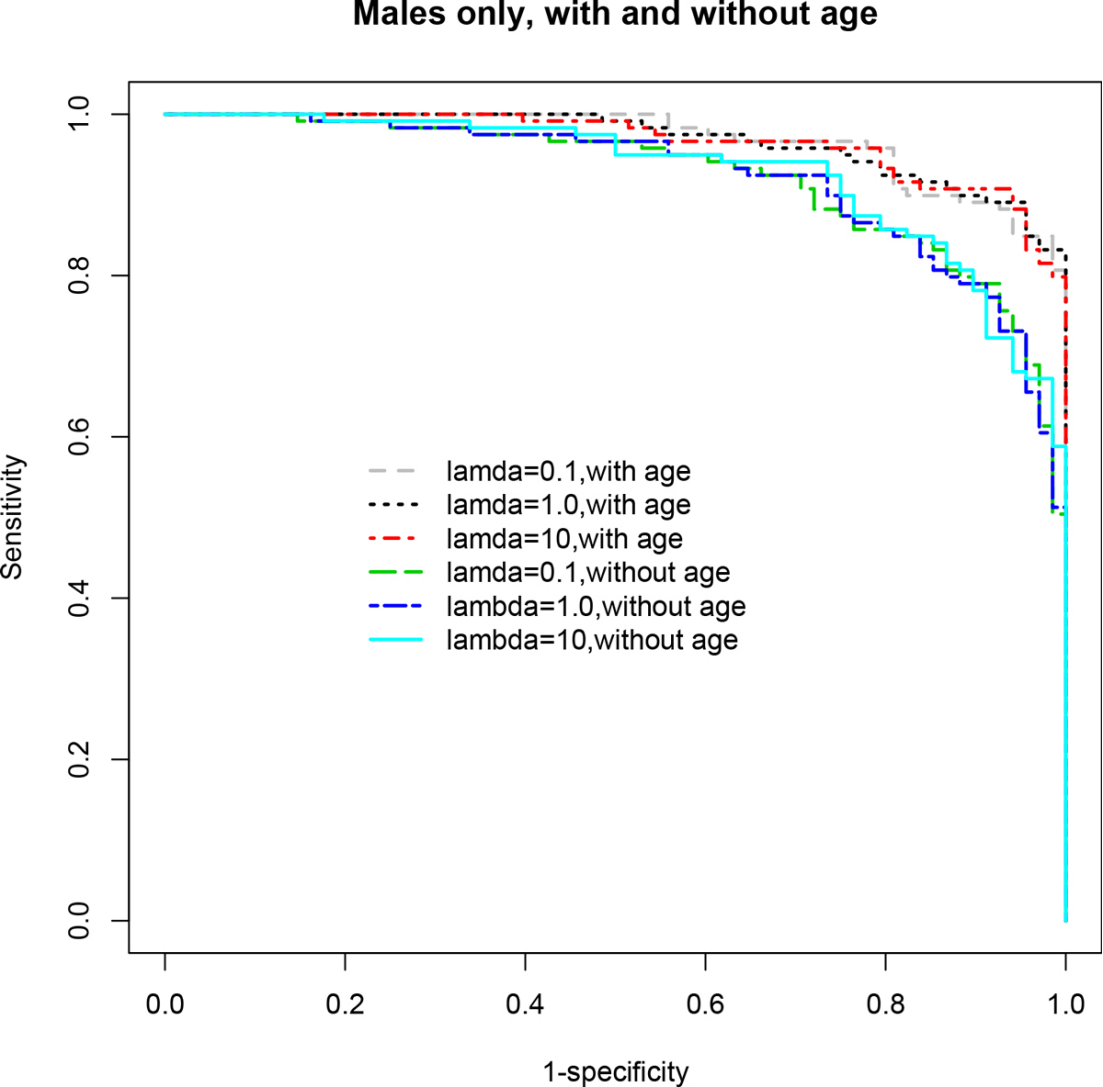
**ROC curves based on multivariable stepwise penalized logistic regression models (*stepPLR*) using the stratified male-only subset**. The age-adjusted final model for λ = 0.1 showed the best performance in terms of AUC. A clear distinction is seen in the ROC curves for age-adjusted models compared to age-unadjusted models. Age-adjusted models demonstrated superior performance overall across all choices of λ. See Table 1 for detailed results and the text for discussion of these results.

**Figure 2:**
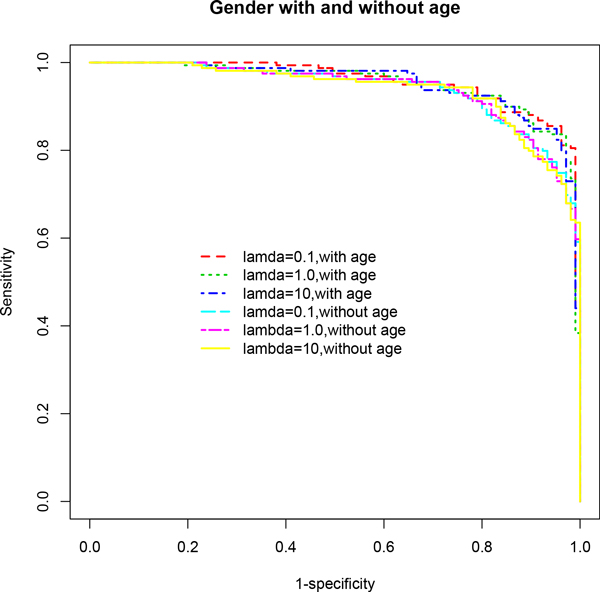
**ROC curves based on multivariable stepwise penalized logistic regression models (*stepPLR*) adjusting for gender effect**. Models that are also adjusted for age effect outperformed those that did not control for age, across all choices of the parameter λ. The age-adjusted final model for λ = 0.1 showed the best performance in terms of AUC. See Table 1 for detailed results and the text for discussion of these results.

**Figure 3:**
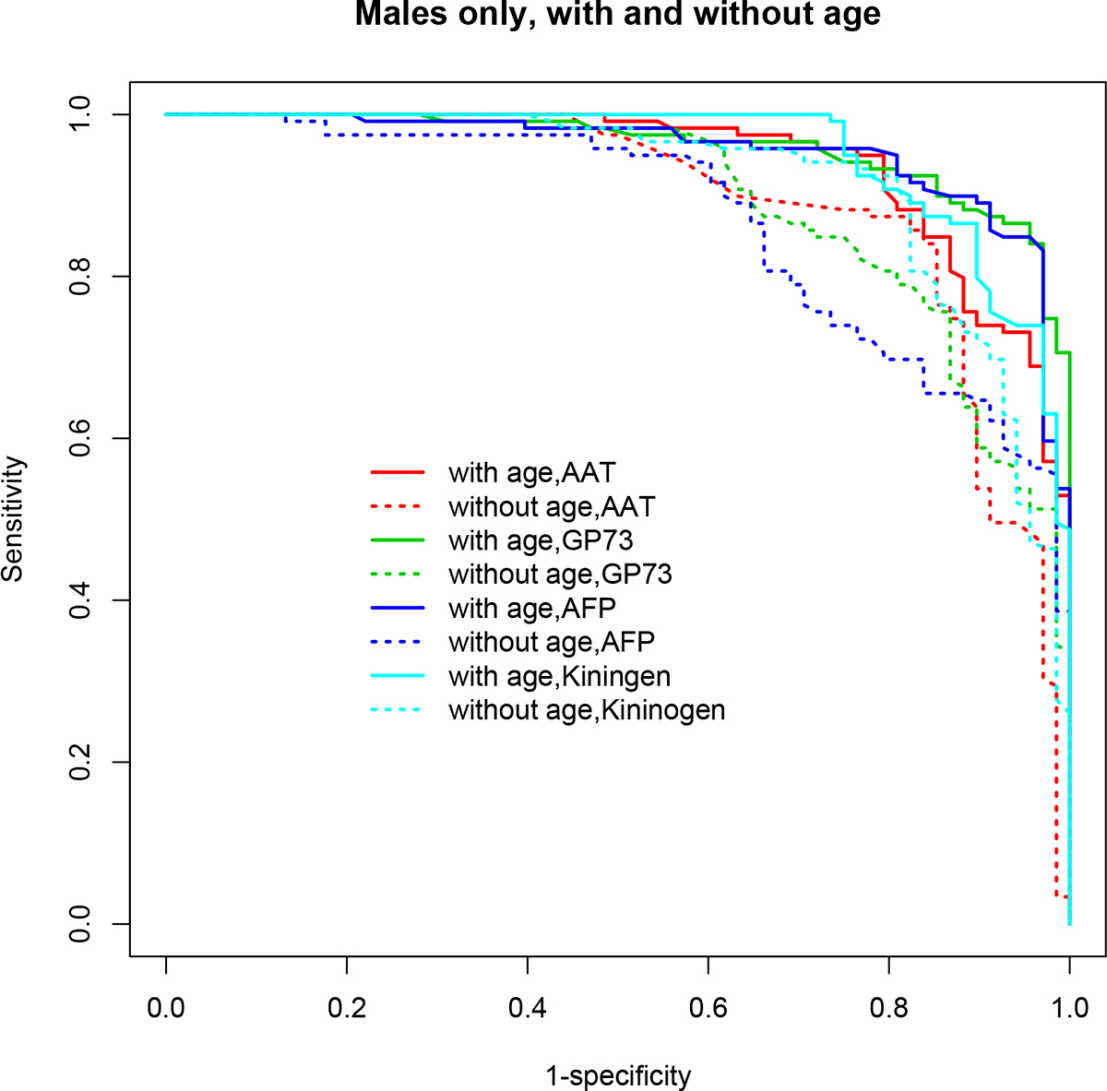
**ROC curves based on multivariable model-based CART analyses (*mob*) using the stratified male-only subset**. Age-adjusted models demonstrated superior performance in terms of AUC. A clear distinction is seen in the ROC curves for age-adjusted models (solid lines) compared to age-unadjusted models (dotted lines). See Table 1 for detailed results and the text for discussion of these results.

**Figure 4:**
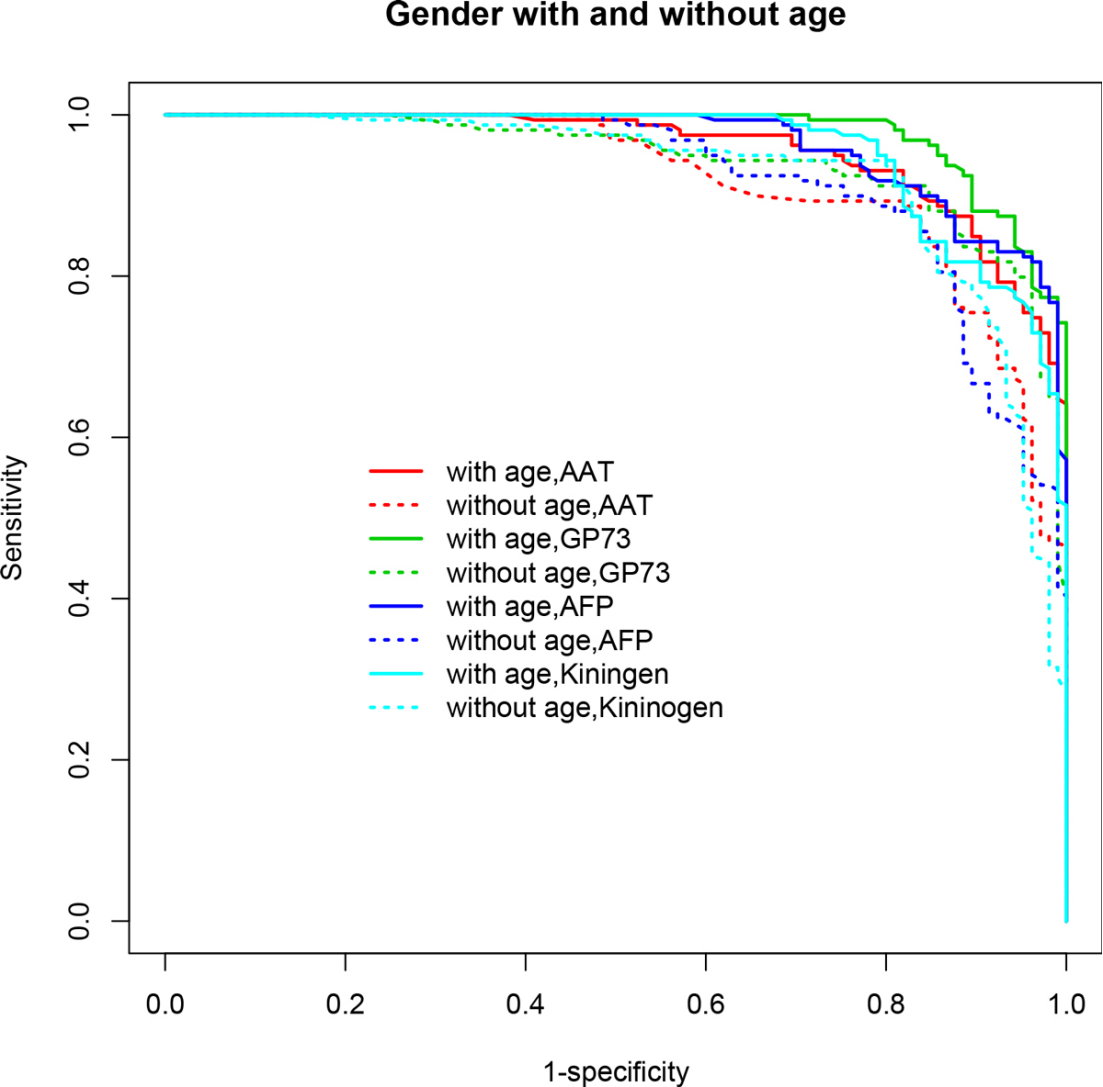
**ROC curves based on multivariable model-based CART analyses (*mob*) incorporating gender and/or age**. Age-adjusted models demonstrated superior performance in terms of AUC when gender effect is accounted for in each model. A clear distinction is seen in the ROC curves for age-adjusted models (solid lines) compared to age-unadjusted models (dotted lines). Table 1 lists the performance measures for these models. A detailed discussion of the results is provided in the text.

**Figure 5:**
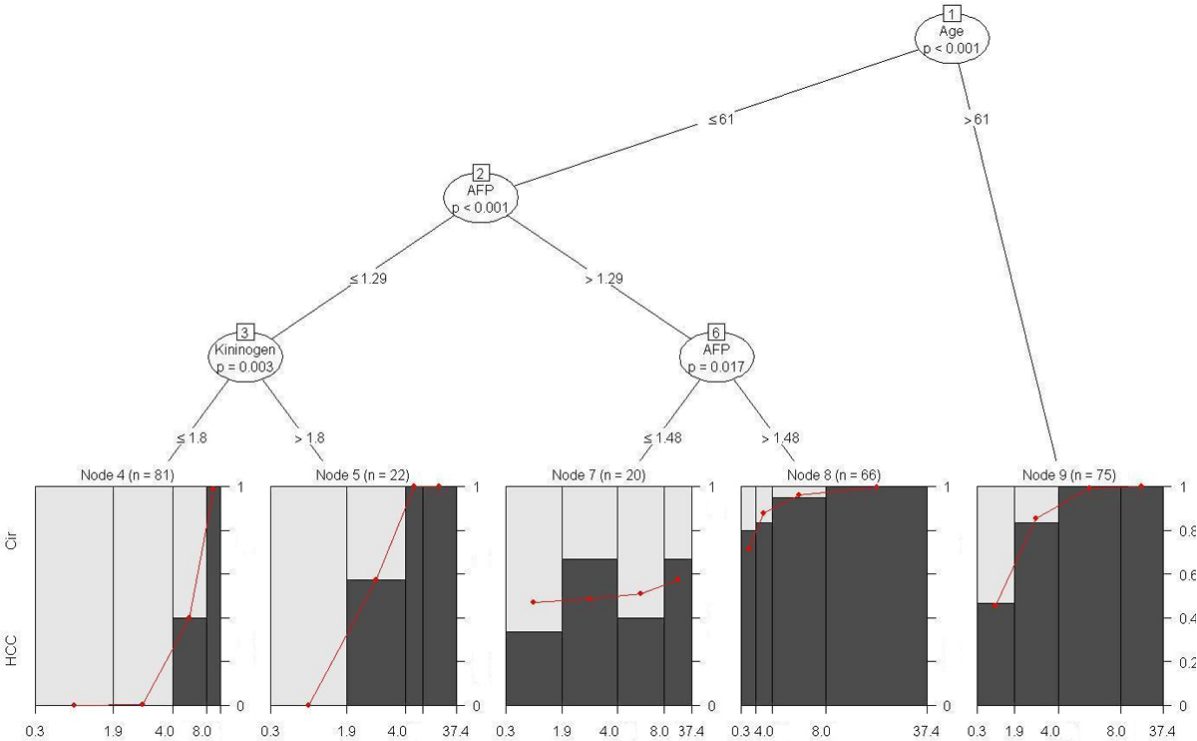
**Model-based CART analysis based on the biomarker GP73 conditional on the tree analysis using AFP, AAT, Kininogen and age, after controlling for site, for the complete data set**. Variables that appear in the tree were involved in a statistically significant split (based on p-value < 0.05). Any two (or more) bins that appear at the bottom child nodes in this tree sharing the same mother node represent disjoint sub-groups of patients identified by this method to be (statistically) significantly different. The sub-groups are defined by the respective cut-points for biomarker levels and age. For example, when gender effect is controlled for in the model it is evident that age alone, independent of other biomarkers, plays a significant role in the incidence of HCC (*p *< 0.001). The node pair (1,9) represents the sub-group of 75 patients older than 61 years that have a significantly higher incidence of HCC compared to younger patients. It provides a unique, visual representation of complex interactions between biomarkers, age and gender though gender is not found to be statistically significant in any of the interactions. In addition, this approach identifies potential cut-points for biomarker levels that are significantly associated with the incidence of HCC. A detailed interpretation of this tree is provided in the text.

**Figure 6:**
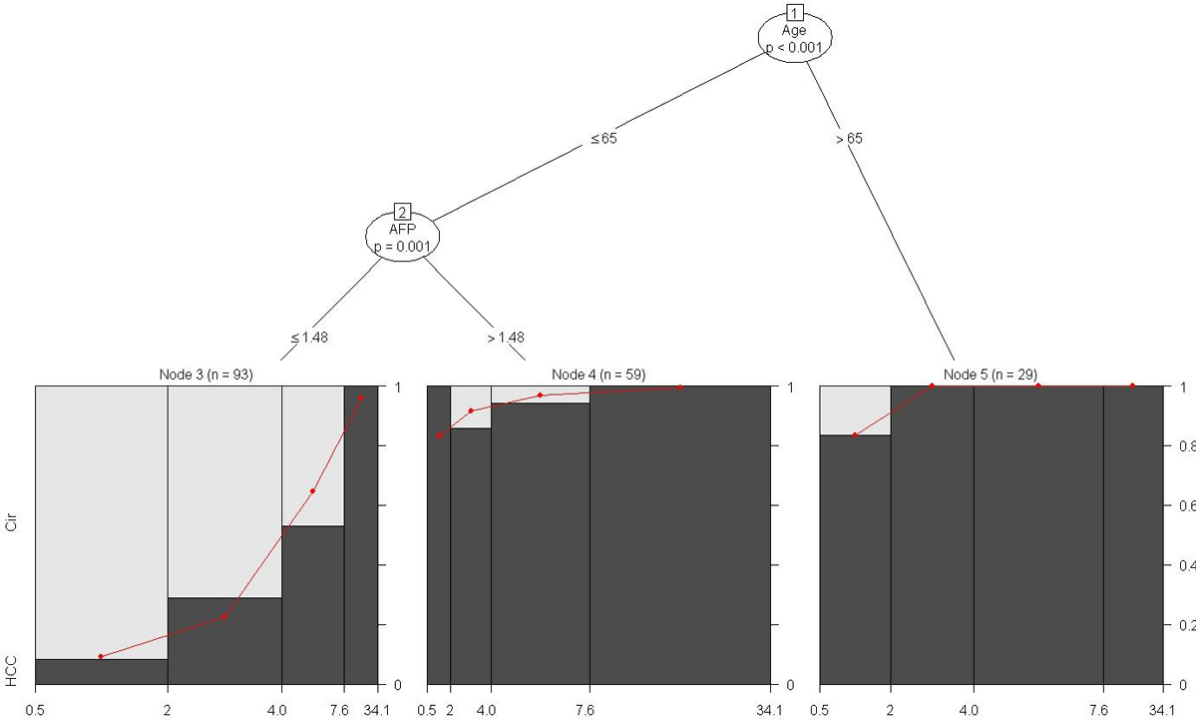
**Model-based CART analysis based on the biomarker GP73 conditional on the tree analysis using AFP, AAT, Kininogen and age, after controlling for site, for the stratified male-only subset**. For this subset, age alone, independent of other biomarkers, plays a significant role in the incidence of HCC (*p *< 0.001). The node pair (1,5) represents the sub-group of 29 men aged >65 that have a significantly higher incidence of HCC. The node pairs (1,2) and (2,4) represent the sub-group of 59 men aged 65 or younger for whom a higher AFP level (>1.48) is significantly associated with increased incidence of HCC (p = 0.001) irrespective of GP73 level. A detailed interpretation of this tree is provided in the text.

**Figure 7:**
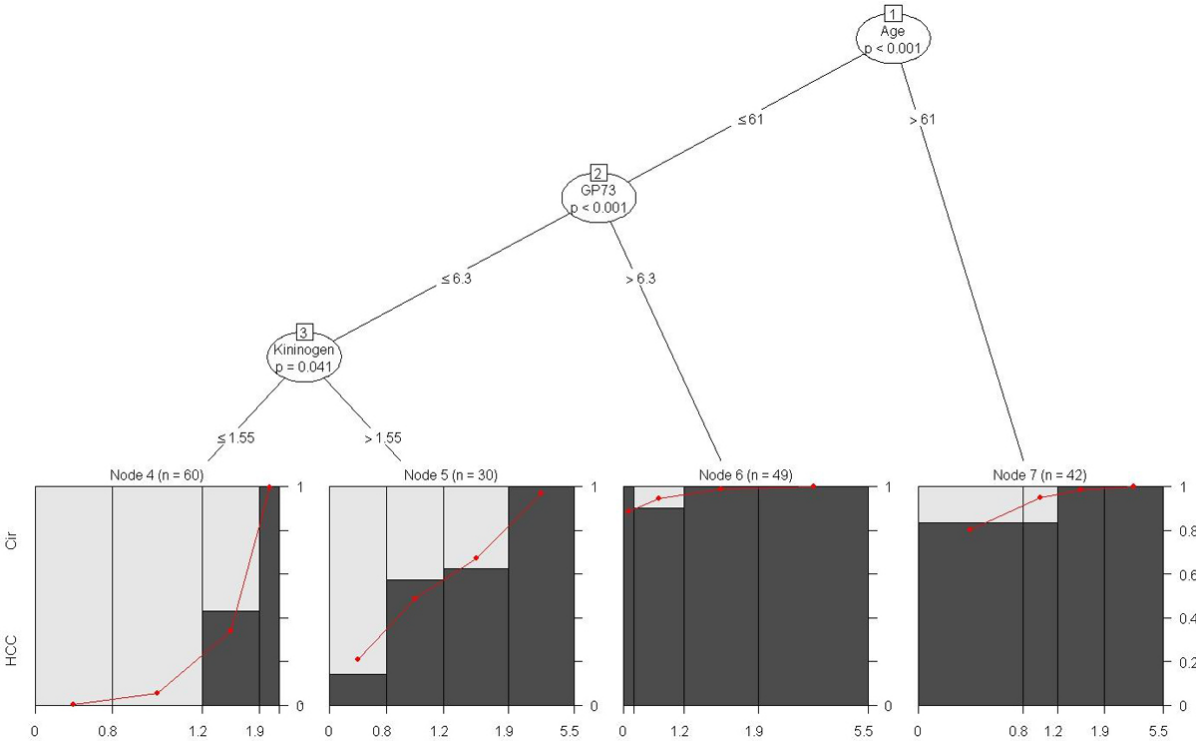
**Model-based CART analysis based on the biomarker AFP conditional on the tree analysis using GP73, AAT, Kininogen and age, after controlling for site, for the stratified male-only subset**. Once again, age alone plays a significant role in the incidence of HCC (*p *< 0.001). The node pair (1,7) represents the sub-group of 42 men aged >61 that have a significantly higher incidence of HCC. This is consistent with the finding based on the gender-adjusted model shown in Figure 5. For men 61 years of age or younger, a higher level of GP73 (>6.3) is significantly associated with increased HCC incidence (*p *< 0.001) independent of AFP and AAT levels. The node pair (2,6) represents this sub-group of 49 men. A detailed interpretation of this tree is provided in the text.

Data obtained across two sites were used in the analyses. In order to adjust for any potential differences in biomarker levels obtained at different sites, a dichotomous, nominal variable site (indicating the site where the data was obtained for each observation) was incorporated into the modeling as a covariate. For each statistical method used, four different models were considered based on the inclusion of age and gender in multivariable analysis. These are listed in Table 1. The stratified dataset consisting of males only (with or without age) was of particular importance due to the known higher incidence of HCC in male patients [2]. Results from multivariable analyses (presented in Tables 1, 2, 3 Figures 1, 2, 3, 4, 5, 6, 7) were compared with those from univariate LR (presented in Tables 1A, B and C, Figure 1 of [26]) and multivariable LR, PLR and CART analyses (presented in Tables 2, 3, 4, Figures 2, 3, 4, 5 of [26]) reported in our previous study [26].

**Table 1:** Model-based performance measures (Multivariable models)

Method (Model)^1^	AUC (95% CI) ^2^	ACC^3^	PPV^4^	NPV^5^	AIC^6^
stepPLR (λ = 0.1) (male only, with age)	0.96(0.94-0.98)	87.71	90.68	82.61	93.92
stepPLR (λ = 1) (male only, with age)	0.96(0.94-0.98)	89.30	92.31	84.29	94.84
stepPLR (λ = 10) (male only, with age)	0.96(0.94-0.98)	89.83	93.10	84.51	95.39
stepPLR (λ = 0.1) (male only, without age)	0.92(0.88-0.95)	83.42	88.59	75.34	135.39
stepPLR (λ = 1) (male only, without age)	0.92(0.88-0.95)	84.49	90.17	76.00	136.39
stepPLR (λ = 10) (male only, without age)	0.92(0.89-0.96)	84.49	90.90	75.32	134.57
stepPLR (λ = 0.1) (gender, with age)	0.95(0.93-0.98)	88.25	91.02	84.25	142.14
stepPLR (λ = 1) (gender, with age)	0.95(0.93-0.98)	88.63	91.61	84.40	142.48
stepPLR (λ = 10) (gender, with age)	0.95(0.93-0.97)	89.01	92.20	84.54	148.32
stepPLR (λ = 0.1) (gender, without age)	0.94(0.91-0.96)	85.61	89.54	80.18	173.22
stepPLR (λ = 1) (gender, without age)	0.94(0.91-0.97)	85.61	90.06	79.65	174.15
stepPLR (λ = 10) (gender, without age)	0.93(0.91-0.96)	85.23	90.54	78.44	180.61
mob(AAT|~.) with age, male only)	0.94(0.91-0.97)	87.29	89.65	83.07	131.68
mob(GP73|~.) with age, male only)	0.95(0.93-0.98)	87.87	91.89	81.42	108.96
mob(AFP|~.) with age, male only)	0.96(0.94-0.98)	89.50	90.67	87.30	103.38
mob(Kin|~.) with age, male only)	0.95(0.92-0.98)	86.74	88.24	83.87	117.63
mob(AAT|~.) without age, male only)	0.88(0.83-0.94)	84.49	87.50	79.10	157.50
mob(GP73|~.) without age, male only)	0.89(0.85-0.94)	80.21	83.61	73.84	153.01
mob(AFP|~.) without age, male only)	0.86(0.81-0.91)	79.14	81.74	73.77	169.15
mob(Kin|~.) without age, male only)	0.91(0.87-0.95)	88.23	89.43	85.93	140.17
mob(AAT|~.) with age, gender)	0.95(0.93-0.97)	87.50	90.38	83.33	171.96
mob(GP73|~.) with age, gender)	0.97(0.96-0.99)	90.90	91.41	90.09	124.26
mob(AFP|~.) with age, gender)	0.96(0.94-0.98)	87.87	89.93	84.76	159.88
mob(Kin|~.) with age, gender)	0.95(0.93-0.97)	89.01	88.23	90.42	155.18
mob(AAT|~.) without age, gender)	0.91(0.88-0.95)	85.98	88.13	82.69	197.87
mob(GP73|~.) without age, gender)	0.94(0.91-0.96)	85.33	92.36	78.06	177.11
mob(AFP|~.) without age, gender)	0.92(0.89-0.95)	85.22	87.50	81.73	190.61
mob(Kin|~.) without age, gender)	0.92(0.88-0.95)	87.87	88.48	86.86	192.78

1. Method utilized for analysis, stepPLR stepwise penalized logistic regression with or without age and/or gender, mob model-based CART, classification and regression trees developed with or without age and/or gender; 2) AUC, area under the curve; 3) ACC, prediction accuracy. 4) PPV, Positive predicative value; 5) NPV, negative predictive value; 6) AIC, Akaike Information Criterion

**Table 2:** Performance measures based on cross-validation (Multivariable Models)

Method (Model)^1^	LOOCV AUC (95% CI)^2^	LOOCV ACC^3^	3CV AUC (95% CI)^4^	3CV ACC (SD)^5^
stepPLR (λ = 0.1) (male only, with age)	0.95(0.93-0.98)	86.63	0.94(0.94-0.95)	86.03(4.78)
stepPLR (λ = 1) (male only, with age)	0.95(0.93-0.98)	87.16	0.95(0.95-0.96)	87.12(4.31)
stepPLR (λ = 10) (male only, with age)	0.94(0.92-0.97)	86.63	0.95(0.94-0.95)	87.49(4.31)
stepPLR (λ = 0.1) (male only, without age)	0.90(0.86-0.94)	80.35	0.90(0.89-0.90)	80.97(5.16)
stepPLR (λ = 1) (male only, without age)	0.89(0.84-0.93)	81.28	0.89(0.89-0.90)	80.71(5.09)
stepPLR (λ = 10) (male only, without age)	0.91(0.87-0.95)	83.96	0.91(0.90-0.91)	82.17(5.37)
stepPLR (λ = 0.1) (gender, with age)	0.95(0.92-0.97)	88.25	0.95(0.94-0.95)	87.96(3.20)
stepPLR (λ = 1) (gender, with age)	0.95(0.92-0.97)	88.25	0.95(0.94-0.95)	88.34(3.51)
stepPLR (λ = 10) (gender, with age)	0.94(0.92-0.97)	88.25	0.95(0.94-0.95)	88.31(3.39)
stepPLR (λ = 0.1) (gender, without age)	0.92(0.88-0.95)	84.84	0.92(0.91-0.92)	84.35(4.23)
stepPLR (λ = 1) (gender, without age)	0.92(0.89-0.95)	84.84	0.92(0.92-0.92)	84.59(4.09)
stepPLR (λ = 10) (gender, without age)	0.92(0.89-0.95)	83.71	0.92(0.91-0.92)	83.72(4.29)
mob(AAT|~.) with age, male only)	0.85(0.79-0.91)	83.43	0.81(0.80-0.82)	76.23(5.71)
mob(GP73|~.) with age, male only)	0.88(0.82-0.93)	83.98	0.87(0.87-0.88)	80.77(4.99)
mob(AFP|~.) with age, male only)	0.86(0.80-0.92)	81.22	0.87(0.87-0.88)	83.66(5.52)
mob(Kin|~.) with age, male only)	0.92(0.88-0.96)	86.19	0.86(0.85-0.86)	79.36(5.08)
mob(AAT|~.) without age, male only)	0.78(0.71-0.85)	75.93	0.82(0.81-0.83)	75.33(5.15)
mob(GP73|~.) without age, male only)	0.83(0.76-0.89)	76.47	0.86(0.85-0.86)	77.81(4.16)
mob(AFP|~.) without age, male only)	0.80(0.74-0.86)	72.72	0.83(0.82-0.84)	76.54(5.42)
mob(Kin|~.) without age, male only)	0.88(0.83-0.93)	85.03	0.86(0.85-0.87)	78.88(4.92)
mob(AAT|~.) with age, gender)	0.87(0.83-0.92)	82.57	0.86(0.78-0.94)	81.19(4.59)
mob(GP73|~.) with age, gender)	0.93(0.89-0.96)	87.50	0.91(0.90-0.91)	84.61(4.03)
mob(AFP|~.) with age, gender)	0.90(0.86-0.94)	83.33	0.89(0.88-0.89)	82.95(4.42)
mob(Kin|~.) with age, gender)	0.89(0.85-0.93)	83.33	0.89(0.88-0.89)	82.81(4.14)
mob(AAT|~.) without age, gender)	0.84(0.79-0.89)	84.46	0.87(0.86-0.87)	80.93(3.71)
mob(GP73|~.) without age, gender)	0.91(0.87-0.94)	83.33	0.89(0.88-0.89)	81.22(4.35)
mob(AFP|~.) without age, gender)	0.87(0.82-0.91)	82.95	0.87(0.87-0.87)	80.60(3.73)
mob(Kin|~.) without age, gender)	0.88(0.84-0.92)	84.85	0.89(0.89-0.90)	83.05(3.97)

1. Method utilized for analysis, stepPLR stepwise penalized logistic regression with or without age and/or gender, mob model-based CART, classification and regression trees developed with or without age and/or gender; 2) LOOCV AUC, leave one out cross validation area under the curve; 3) LOOCV ACC, leave one out cross validation prediction accuracy; 4) 3CV AUC, three fold cross validation area under the curve; 5) 3CV ACC, three fold cross validation prediction accuracy.

**Table 3:** Sensitivities and Specificities (Multivariable Models)

Method (Model)^1^	Sensitivity (95% CI)	Sensitivity (LOOCV)^3^	Specificity (95% CI)^4^	Specificity (LOOCV)
stepPLR (λ = 0.1) (male only, with age)	89.92(85.69-95.80)	89.08	83.82(73.53-91.18)	82.35
stepPLR (λ = 1) (male only, with age)	90.76(86.55-95.81)	89.92	86.76(77.94-94.12)	82.35
stepPLR (λ = 10) (male only, with age)	90.75(86.56-95.81)	88.24	88.24(75.00-92.65)	83.82
stepPLR (λ = 0.1) (male only, without age)	84.87(79.83-90.76)	84.03	80.88(70.59-89.71)	79.41
stepPLR (λ = 1) (male only, without age)	84.82(78.15-90.76)	83.19	83.82(75.00-92.65)	77.94
stepPLR (λ = 10) (male only, without age)	84.03(78.15-91.60)	84.03	85.29(76.47-92.65)	83.82
stepPLR (λ = 0.1) (gender, with age)	89.94(84.91-94.39)	89.94	86.67(80.00-92.38)	83.81
stepPLR (λ = 1) (gender, with age)	90.57(86.16-94.94)	89.31	86.67(80.00-93.33)	86.66
stepPLR (λ = 10) (gender, with age)	89.31(84.28-93.71)	88.68	88.57(81.90-94.29)	88.58
stepPLR (λ = 0.1) (gender, without age)	86.16(86.50-91.19)	84.91	84.76(77.14-91.43)	84.76
stepPLR (λ = 1) (gender, without age)	85.53(79.87-90.57)	84.90	85.71(80.00-93.33)	84.76
stepPLR (λ = 10) (gender, without age)	84.27(79.87-90.57)	81.76	86.66(80.00-92.38)	86.66
mob(AAT|~.) with age, male only)	90.43(79.36-95.63)	86.96	81.82(73.45-88.33)	77.27
mob(GP73|~.) with age, male only)	88.69(77.51-94.61)	86.08	86.36(78.39-91.83)	80.30
mob(AFP|~.) with age, male only)	93.04(83.20-98.32)	84.34	83.33(75.41-89.75)	75.76
mob(Kin|~.) with age, male only)	91.30(81.26-96.59)	86.96	78.79(67.71-83.92)	84.85
mob(AAT|~.) without age, male only)	88.23(82.35-93.28)	78.15	77.94(67.65-86.76)	72.06
mob(GP73|~.) without age, male only)	85.71(79.83-92.44)	81.51	70.59(60.29-80.88)	67.64
mob(AFP|~.) without age, male only)	86.55(80.67-92.44)	79.83	66.17(54.41-76.47)	60.29
mob(Kin|~.) without age, male only)	92.43(87.39-96.64)	90.75	80.88(70.59-89.71)	75.00
mob(AAT|~.) with age, gender)	89.33(84.28-93.71)	86.16	85.71(79.05-92.38)	77.14
mob(GP73|~.) with age, gender)	94.97(91.82-98.11)	92.45	86.68(80.00-92.38)	80.00
mob(AFP|~.) with age, gender)	91.19(86.75-94.97)	88.05	83.81(76.19-90.48)	76.19
mob(Kin|~.) with age, gender)	94.39(90.54-97.48)	89.30	80.95(73.33-80.95)	74.28
mob(AAT|~.) without age, gender)	88.67(84.28-93.71)	87.42	81.90(74.29-88.57)	80.00
mob(GP73|~.) without age, gender)	83.64(77.36-89.31)	82.39	89.52(83.81-95.24)	84.76
mob(AFP|~.) without age, gender)	88.05(83.65-93.08)	87.42	80.95(73.33-88.65)	76.19
mob(Kin|~.) without age, gender)	91.82(88.05-96.45)	89.93	81.90(75.24-88.60)	77.14

1. Method utilized for analysis, stepPLR stepwise penalized logistic regression with or without age and/or gender, mob model-based CART, classification and regression trees developed with or without age and/or gender; 2) optimal sensitivity and 95% Confidence interval; 3) Optimal sensitivity following leave one out cross validation; 4) Optimal specificity; 5) Optimal specificity following leave one out cross validation.

It is evident from the results reported in our previous study [26] that univariate LR models performed uniformly worse than multivariable models that utilized multiple biomarkers using *any *of the three methods considered in that study, namely, multivariable LR, PLR and CART. It turned out that the best performing univariate model (GP73) produced a model-based AUC of 0.87 (95% CI (0.84, 0.91)) and ACC of 0.78, a result that fell far short of those of multivariable models, and thus emphasized the need for including multiple biomarkers and additional confounding clinical variables into the model. In addition, among the three multivariable methods considered, PLR and CART outperformed LR. PLR provided the best overall performance while CART served as a useful alternative by providing useful cut-points for biomarker levels. In this study, we improve upon the performance of these two methods by implementing a stepwise PLR (*stepPLR*) and a model-based CART (*mob*) approach, respectively.

In the following paragraphs, the performance of various models is compared for each method and the results summarized and interpreted. Performance measures such as AUC, ACC, PPV, NPV, sensitivity and specificity are compared between age-adjusted and age-unadjusted models when gender effect is considered and also for the stratified male-only subset. With the exception of AUC, which is expressed on the [0,1] scale, each quantity is measured on a [0,100] scale. We re-scale each measure to [0,1] in our comparisons for the sake of uniformity and convenience. Difference between models for each quantity is expressed as actual difference (indicating better or worse performance) and not as relative difference, i.e., a difference of 5 units on the [0,100] scale is equivalent to 5% (or 0.05) on the [0,1] scale.

In particular, *stepPLR *and *mob *showed significant improvements in predictive performance when age was included in the model after adjusting for gender differences compared to the model excluding age (Table 1 Figures 2, 4). *stepPLR *showed a median increase in AUC (ACC) of 2% (3.02%) (across all choices of λ) while *mob *showed a median increase of 4% (2.1%) across the four models considered. When the stratified subset consisting of only males was used in the analysis, this difference increased to 5% (4.81%) and 5.5% (5.22%), respectively, for *stepPLR *and *mob *(Table 1 Figures 1, 3). For this subset, the *mob *model based on the biomarker AFP conditional on the tree analysis using GP73, AAT, Kininogen and age, after controlling for site, resulted in the maximum increase of 10% in AUC and 10.36% in ACC due to the inclusion of age (Table 1).

A considerable increase of 6% in AUC and 7.63% in ACC were also noted due to the inclusion of age in the *mob *model for GP73 conditional on the tree analysis using AFP, AAT, Kininogen and age, after controlling for site. In addition, this model resulted in the maximum increase of 4% in AUC and 5.57% in ACC when controlled for gender effect, among all methods and models considered. These are significant improvements over our previous findings in which all three multivariable methods used (LR, PLR and CART) showed improvements in AUC and ACC in excess of 4% for this data, with PLR (λ = 1) showing the best overall increase in ACC of only about 5% [26]. Consistent with our recent findings [26], a marked improvement was observed in the predictive performance of each method based on this stratified dataset independent of whether age is included in the model. The inclusion of age, however, resulted in the best predictive model across all combinations of methods and models considered (Table 1). In addition, the inclusion of age resulted in a substantial decrease in Akaike Information Criterion (AIC) for *stepPLR *(across all choices of λ) and *mob *(across all models considered) both for the stratified male only dataset and when gender differences are accounted for in the model (Table 1). This finding underscores the significant role played by the variable age in model selection and in the predictive performance of the final model.

Furthermore, PPV and NPV capture other critical aspects of the performance of a model. For our application, PPV represents the proportion of patients *correctly *predicted to have HCC while NPV represents the proportion of patients *correctly *predicted to have cirrhosis. A high PPV means that the model only rarely classifies a HCC patient as having cirrhosis, and is therefore a desirable characteristic in a model. Table 1 lists the best performing models and methods in terms of PPV and NPV. Models that adjusted for age effect generally showed a higher median PPV or NPV compared to those that did not (across all choices of λ and models considered), a result consistent with our previous findings [26]. A significantly higher increase in NPV was observed in models adjusting for age, compared to PPV, using both methods (median increase of 4.75% and 8.29% for *stepPLR*, and 3.3% and 5.78% for *mob*, respectively, when adjusted for gender effect and in the stratified male-only subset). For the stratified male only subset, *mob *improved PPV by 5.22% with the inclusion of age. The *mob *model based on AFP conditional on the tree analysis using GP73, AAT, Kininogen and age, after controlling for site, resulted in the maximum improvement in PPV of 8.93% and in NPV of 13.53% due to the inclusion of age for this subset. These compare with maximum increases of 3.57% for PPV (LR and PLR, λ = 0.1) and 9% for NPV (PLR, λ = 10) from our previous study [26]. When gender effect was adjusted for, the above *mob *model also showed the maximum increase in PPV (2.43%) due to the inclusion of age. On the other hand, *stepPLR *(λ = 10) resulted in the maximum increase in NPV of 6.1% compared to the 4.7% maximum increase achieved by PLR (λ = 10) in our previous study [26]. The highest PPV (93.1%) was achieved for the stratified male only data by *stepPLR *(λ = 10) across both methods and all models considered, also an improvement over the maximum 91.96% achieved in our previous study [26].

In terms of model-based sensitivity and specificity, both *stepPLR *and *mob *produced an improvement due to the inclusion of age in stratified male only data. *stepPLR *showed the highest overall increase in sensitivity (median increase of 5.94% across choices of λ) while *mob *showed the highest overall increase in specificity (median increase of 9.83% across all models considered). Once again, the *mob *model based on the biomarker AFP resulted in the maximum improvement in both sensitivity (6.49%) and specificity (17.16%) due to the inclusion of age for this subset. In comparison, LR and PLR (λ = 10) produced the greatest improvement (of over 6% each) in our previous study [26]. Model-based and cross-validation based sensitivities and specificities are displayed in Table 3. When gender effect was adjusted for in the model, a more sensitive model (increase of 5.04%) was afforded by *stepPLR *(λ = 1,10) while *mob *provided a more specific (increase of 3.81%) model due to the inclusion of age (Table 1).

### Predictive performance of multivariable models using cross-validation

While model based metrics such as AUC, ACC, PPV and NPV provide a measure of the predictive performance of a model, equivalent versions of these quantities based on cross-validation are based on blinded, independent datasets and therefore provide the true predictive performance of the model. Table 2 presents the AUC (with 95% CI) and ACC for each model and method used based on LOOCV and 3CV. A considerable improvement in AUC was observed in models that included age across both methods for the stratified male only data. The median value of this increase was 5.5% for AUC based on LOOCV and 4% for AUC based on 3CV. When gender is accounted for in the model, the inclusion of age also results in an improvement in AUC of 3% for *stepPLR *(median value across choices of λ, based on both LOOCV and 3CV) and a 2.5% increase for *mob*. In terms of prediction accuracy, a significant improvement in ACC was observed in models that included age for both *stepPLR *and *mob *for the stratified male only data. For *stepPLR*, the median value of this increase was around 5.88% for ACC based on LOOCV and around 5.32% for ACC based on 3CV. For *mob*, the median value of this increase was around 7.51% for ACC based on LOOCV and around 1.93% for ACC based on 3CV. The *mob *model based on AFP conditional on the tree analysis using GP73, AAT, Kininogen and age, after controlling for site showed increases of 8.5% and 7.12% in ACC based on LOOCV and 3CV, respectively. When gender effect was accounted for in the model, the inclusion of age also resulted in improvements of 3.41% and 3.75% for *stepPLR *(median value across choices of λ) based on LOOCV and 3CV, respectively. On the other hand, the performance of *mob *was observed to vary between models and cross-validation methods. These results show an overall improvement in the predictive performance of models based on *stepPLR *and *mob *over our previous findings based on multivariable LR, PLR and CART [26].

### Interpretation of model-based CART (*mob*) results

Multivariable model-based CART (*mob*) analyses revealed several different and interesting aspects of the data that more traditional methods such as multivariable LR and PLR are not capable of exposing. To a lesser extent, methods like *stepPLR *and CART (ctree) also suffer from this issue. Four different statistical models, one for each biomarker conditional on the tree analysis based on the remaining biomarkers, age and/or gender (controlling for site) were considered in this analysis. As noted earlier, *mob *combines a parametric approach based on generalized linear models with CART. In this case, the outcome variable is binary, i.e., whether a patient has HCC or not, and hence the parametric method of choice is logistic regression.

The model based on the biomarker GP73 conditional on the tree analysis using AFP, AAT, Kininogen and age, after controlling for site, showed excellent performance in terms of cross-validated measures, particularly when gender effect was also included (Table 2). This model also showed a substantial improvement in AUC and ACC due to the inclusion of age. First, we will use this model to illustrate *mob *results. Figures 5 and 6 graphically represent the results for this model when controlling for gender effect and for the stratified male only subset, respectively. When gender effect is controlled for in the model it is evident (from Figure 5) that age alone, independent of other biomarkers, plays a significant role in the incidence of HCC (*p *< 0.001) (*n *= 75 patients corresponding to node pair (1,9) in Figure 5). Older patients (>61) are at an increased risk of HCC incidence. Among those aged 61 or younger, higher level of AFP (>1.48) is significantly associated with increased incidence of HCC (*p *= 0.017) irrespective of GP73 level (*n *= 66 patients corresponding to node pairs (1,2), (2,6) and (6,8)). The subgroup of 20 patients aged 61 or lower whose AFP level lies in the range (1.29,1.48] represents varying incidence of HCC depending on GP73 level. Finally, younger patients with lower levels of AFP (age <= 61, AFP <= 1.29) represent a subgroup whose HCC incidence significantly increases with higher GP73 and Kininogen levels (GP73>4.035 and Kininogen>1.8).

For the stratified male only subset it is evident (from Figure 6) that age alone, independent of other biomarkers, plays a significant role in the incidence of HCC (*p *< 0.001) (*n *= 29 men corresponding to node pair (1,5) in Figure 6). Older men (>65) are at an increased risk of HCC incidence. Among those aged 65 or younger, higher level of AFP (>1.48) is significantly associated with increased incidence of HCC (*p *= 0.001) irrespective of GP73 level (*n *= 59 men corresponding to node pairs (1,2) and (2,4)). Even among younger men with lower levels of AFP (age <= 65, AFP <= 1.48), the incidence of HCC increases with higher GP73 levels (*n *= 93 corresponding to node pair (2,3)) as indicated by the increasing red curve.

In terms of overall and consistent improvement in performance (evaluated by the various measures) due to the inclusion of age, the *mob *model based on AFP conditional on the tree analysis using GP73, AAT, Kininogen and age (after controlling for site) is the best performer for this subset. Figure 7 graphically illustrates this model. Once again, age alone plays a significant role in the incidence of HCC (*p *< 0.001) (*n *= 42 men corresponding to node pair (1,7) in Figure 7). Older men (>61) are at an increased risk of HCC incidence. This is consistent with the finding based on the gender-adjusted model shown in Figure 5. For men 61 years of age or younger, a higher level of GP73 (>6.3) is significantly associated with increased HCC incidence (*p *< 0.001) independent of AFP and AAT levels (*n *= 49 men corresponding to node pair (2,6) in Figure 7). However, men with a lower GP73 (<= 6.3) and higher Kininogen (>1.55) levels in this subgroup have an increased incidence of HCC with higher levels of AFP (*n *= 30 men corresponding to node pairs (2,3) and (3,5) in Figure 7). This is indicated by the steep increasing red line below node 5 in Figure 7.

## Conclusions

HCC, like many cancers, is characterized by a large degree of heterogeneity. This makes the detection of cancer by serum biomarkers difficult, which results in late detection and poor outcome. With the large degree of genetic heterogeneity, it is generally assumed that no single serum biomarker will be able to detect all cases of HCC. Currently, serum levels of AFP are used in combination with several imaging methodologies to identify HCC. However, the clinical usefulness of AFP is limited by the poor sensitivity of this marker. That is, AFP is elevated in only 60-70% of individuals with HCC. It is important to note that genetically, AFP negative cancers are thought to be fundamentally different than AFP positive tumors. Thus, the detection of serum AFP is useful in the detection of a specific type of HCC. However, it is assumed that multiple markers will be required for the detection of all cases of HCC.

In this paper, we demonstrated the usefulness of incorporating multiple biomarkers and relevant clinical variables into a statistical model for predicting the incidence of HCC. We built on the foundation provided by our recent work [26] and investigated the predictive performance of two different yet related methods, namely *stepPLR *and *mob*, in distinguishing HCC patients from cirrhotic patients. These two methods are improvisations of PLR and CART discussed in our previous study, the former incorporating stepwise model selection in PLR and the latter incorporating a model-based approach to CART. Both these approaches provided significantly improved results not only compared to the use of single and multiple biomarkers (univariate and multivariable LR) but also compared to those based on their counterparts, PLR and CART. A novel aspect of our previous approach was the application of CART for analyzing and interpreting biomarker data for HCC. This non-parametric approach is a useful alternative to traditional parametric methods like LR and PLR that automatically incorporates interactions between multiple biomarkers and/or clinical variables. The extension of this approach using *mob *in this paper borrows strength from the binary recursive partitioning approach in CART as well as the parametric approach in traditional multivariable LR and is based on generalized linear models. This is reflected in the significantly improved predictive performance of this method relative to those based on PLR and CART presented in our recent study [26]. This flexible modeling approach provided potentially useful cut-offs for biomarkers and clinical variables alike that indicated a statistically significant association with increased HCC incidence in an interpretable and systematic manner. The two methods outlined in this paper can be seen as complementary to PLR and CART and it sets the stage for further evaluation and validation of the clinical significance of these results in future, larger studies. An important finding in this study, as in our previous study, is the marked improvement in predictive performance due to the inclusion of clinical factors such as age and gender. This improvement was seen to be independent of the method used in the analysis. The inclusion of other clinical factors such as Alanine transaminase (ALT), Aspartate transaminase (AST) and Alkaline phosphatase (ALK) levels may be able to increase performance even further. This is currently under investigation.

## List of abbreviations used

HCC: hepatocellular carcinoma; HBV: hepatitis B virus; HCV: hepatitis C virus; AFP: alpha-fetoprotein; AFP-L3: core fucosylated glycoform of AFP; GP73: golgi protein; AAL: Aleuria aurantia lectin; US: ultrasound; MRI: magentic resonance imaging; CT: computed tomography; ALT: Alanine transaminase; AST: Aspartate transaminase; ALK: Alkaline phosphatase; LR: logistic regression; PLR: penalized logistic regression; stepPLR: stepwise penalized likelihood regression; CART: classification and regression tree; Mob: model-based classification and regression trees; LOOCV: leave one out cross validation; 3CV: three-fold cross validation; AUC: area under the curve; ROC: receiver operating characteristic; PPV: positive predictive value; NPV: negative predictive value; AIC: Akaike information criterion; ACC: prediction accuracy; CI: confidence interval; Lectin FLISA: lectin fucose linked immunosorbent assay; LMW kininogen: low molecular weight kininogen.

## Authors' contributions

ASM, KD and MW conceived the study. TB, JM and AMD provided patient serum samples. ASM and MW performed the experiments and collected biomarker data. MW analyzed the data under guidance from KD and ASM. KD, ASM and MW interpreted the results and contributed to the writing of the manuscript.

## Competing interests

The authors declare that they have no competing interests.
