# Correction: A comparison of statistical methods for the detection of hepatocellular carcinoma based on serum biomarkers and clinical variables

**DOI:** 10.1186/1755-8794-6-S3-S11

**Published:** 2013-12-20

**Authors:** Mengjun Wang, Anand Mehta, Timothy M Block, Jorge Marrero, Adrian M Di Bisceglie, Karthik Devarajan

**Affiliations:** 1 Drexel University College of Medicine, 3508 Old Easton Rd, Doylestown, PA 18902, USA; 2 Division of Gastroenterology, University of Michigan, 3912 Taubman Center, Ann Arbor, MI 48109, USA; 3 Saint Louis University School of Medicine, 1402 S. Grand FDT 12th Floor, St. Louis, MO 63104, USA; 4 Department of Biostatistics and Bioinformatics, Fox Chase Cancer Center, 333 Cottman Avenue, Philadelphia, PA 18901, USA

## Correction

Our original article was published in *BMC Medical Genomics *in the supplement containing selected articles from the IEEE International Conference on Bioinformatics and Biomedicine 2012 (IEEE BIBM 2012) [1]. After publication, it was noticed that the ROC curves in Figures 1, 2, 3, 4 displayed Sensitivity vs. Specificity rather than Sensitivity vs. 1-Specificity, as labeled. These figures have been reproduced here in the correct format, displaying Sensitivity vs. 1-Specificity, and should replace the corresponding figures in the original article. However, AUC values remain unaffected by this change.

**Figure 1:**
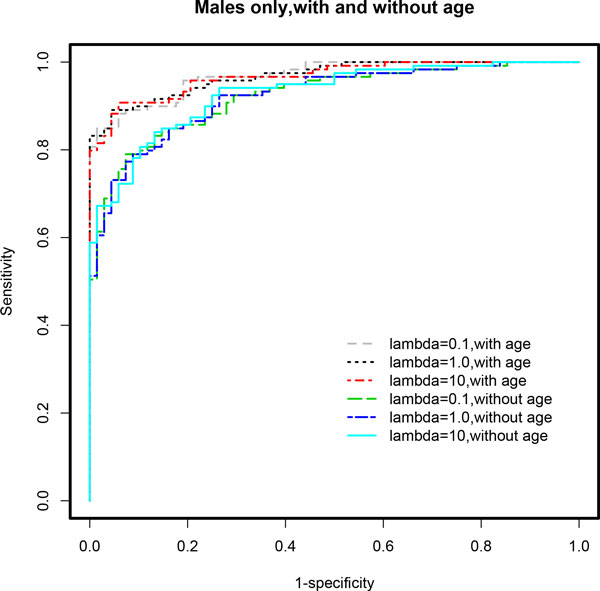
**ROC curves based on multivariable stepwise penalized logistic regression models (*stepPLR*) using the stratified male-only subset**. The age-adjusted final model for λ = 0.1 showed the best performance in terms of AUC. A clear distinction is seen in the ROC curves for age-adjusted models compared to age-unadjusted models. Age-adjusted models demonstrated superior performance overall across all choices of λ. See Table 1 for detailed results and the text for discussion of these results.

**Figure 2:**
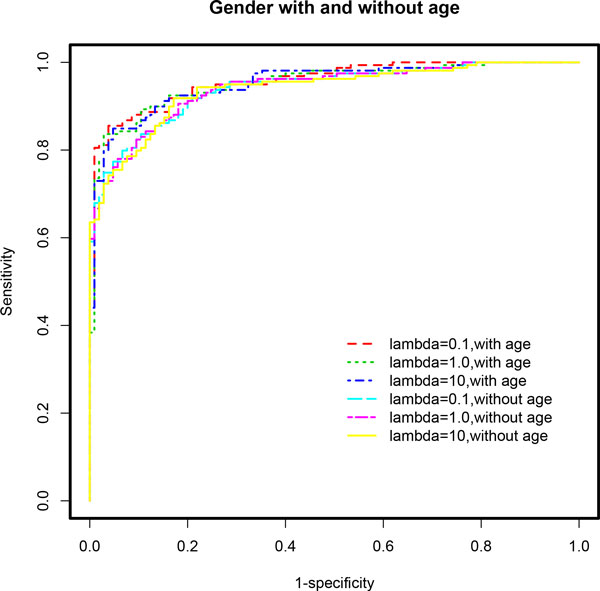
**ROC curves based on multivariable stepwise penalized logistic regression models (*stepPLR*) adjusting for gender effect**. Models that are also adjusted for age effect outperformed those that did not control for age, across all choices of the parameter λ. The age-adjusted final model for λ = 0.1 showed the best performance in terms of AUC. See Table 1 for detailed results and the text for discussion of these results.

**Figure 3:**
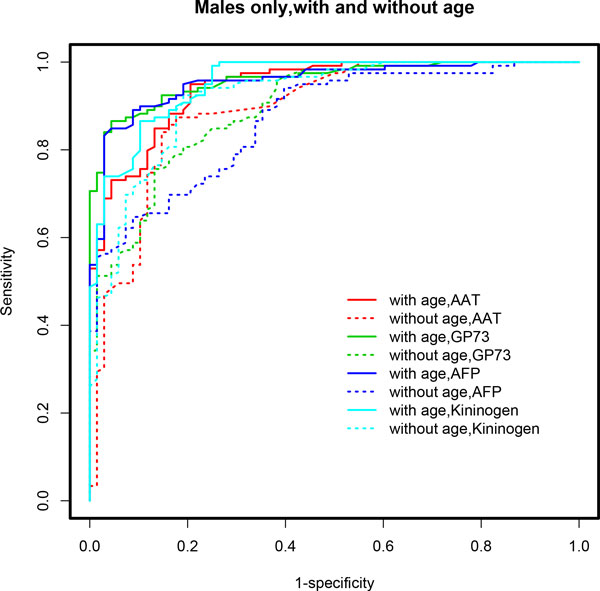
**ROC curves based on multivariable model-based CART analyses (*mob*) using the stratified male-only subset**. Age-adjusted models demonstrated superior performance in terms of AUC. A clear distinction is seen in the ROC curves for age-adjusted models (solid lines) compared to age-unadjusted models (dotted lines). See Table 1 for detailed results and the text for discussion of these results.

**Figure 4:**
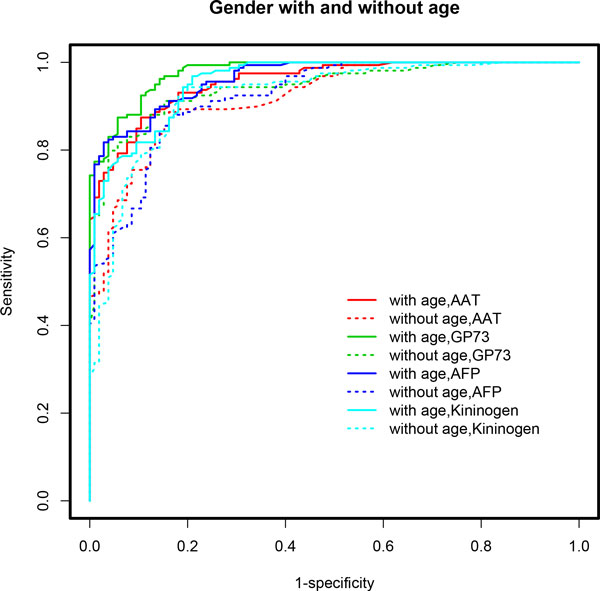
**ROC curves based on multivariable model-based CART analyses (*mob*) incorporating gender and/or age**. Age-adjusted models demonstrated superior performance in terms of AUC when gender effect is accounted for in each model. A clear distinction is seen in the ROC curves for age-adjusted models (solid lines) compared to age-unadjusted models (dotted lines). Table 1 lists the performance measures for these models. A detailed discussion of the results is provided in the text.
